# Synchronized Dual Pulse Gastric Electrical Stimulation Induces Activation of Enteric Glial Cells in Rats with Diabetic Gastroparesis

**DOI:** 10.1155/2014/964071

**Published:** 2014-04-10

**Authors:** Wei Yang, Nian Wang, Xue Shi, Jie Chen

**Affiliations:** Department of Gastroenterology, Union Hospital, Tongji Medical College, Huazhong University of Science and Technology, Wuhan 430022, China

## Abstract

*Objective.* The aims of this study were to investigate the effects of synchronized dual pulse gastric electrical stimulation (SGES) on gastric motility in different periods for diabetic rats and try to explore the possible mechanisms of the effects.* Methods.* Forty-six rats were used in the study. Gastric slow waves were recorded at baseline, 7–14-day diabetes and 56–63-day diabetes before and after stimulation and the age-matched control groups. SGES-60 mins and SGES-7 days (60 mins/day) were performed to test the effects on gastric motility and to evaluate glial marker S100B expression in stomach.* Results.* (1) Gastric emptying was accelerated in 7–14-day diabetes and delayed in 56–63-day diabetes. (2) The S100B expression in 56–63-day diabetes decreased and the ultrastructure changed. (3) The age-associated loss of EGC was observed in 56–63-day control group. (4) SGES was able to not only accelerate gastric emptying but also normalize gastric slow waves. (5) The S100B expression increased after SGES and the ultrastructure of EGC was partially restored. The effect of SGES-7 days was superior to SGES-60 mins.* Conclusions.* Delayed gastric emptying due to the growth of age may be related to the EGC inactivation. The effects of the SGES on gastric motility may be associated with EGC activation.

## 1. Introduction

Gastroparesis is a chronic, symptomatic disorder of the stomach that is characterized by delayed gastric emptying in the absence of mechanical obstruction [1]. This causes digestive difficulties as the food starts to move too slowly through the stomach. The etiology of gastroparesis is diverse and the prevalence is unknown. But it is generally accepted that, in patients with diabetes, gastrointestinal symptoms occur more frequently than in the general population [2]. There are about one-third of cases diagnosed with gastroparesis having diabetes [3]. This situation is common and is increasing all over the world. Symptoms commonly associated with disordered gastric emptying include nausea, retching, vomiting, early satiety, abdominal bloating, loss of appetite, and abdominal pain or abdominal discomfort [4]. Gastrointestinal dysfunction increases the morbidity of diabetes mellitus and affects the quality of life [5].

Diabetic gastroparesis is common but without satisfactory treatment in clinical practice. Gastric electrical stimulation (GES) is a potentially alternative therapy for the medical or surgical treatment of difficult gastroparesis [6–8]. Previous research had shown that synchronized gastric electrical stimulation was able to significantly increase gastric emptying in nonobese mice with diabetic gastroparesis [9]. Meanwhile, a previously performed study by our group had suggested that synchronized gastric electrical stimulation of dual pulses (SGES) with appropriate parameters can accelerate gastric emptying, restore gastric accommodation, and improve gastric slow waves impaired by vagotomy via the nitrergic pathway in dogs [10]. Despite such advantages, the mechanism of action of GES remains poorly understood.

Gastric emptying is mediated via vagovagal reflex pathway. Earlier studies had found that delayed gastric emptying was presented after truncal vagotomy, but there were about one-half of the patients returning to normal one month later; this finding implied that neural pathways other than the truncal vagus compensated impaired motility functions [11]. Additionally, several researches have indicated that the EGC were lost and the expression of glial cell line-derived neurotrophic factor (GDNF) was significantly decreased in diabetes rats [12]. And one study showed that the loss of EGC, but not of interstitial cells of Cajal (ICC) and enteric neurons, was found in the terminal ileum of the patients with severe slow-transit type constipation [13]. Moreover, some findings have demonstrated that EGC can regulate neuronal plasticity [14]. All these findings implied that EGC are not only able to integrate neural network and modulate neuronal activities in enteric nervous system (ENS) but also influence gastric gastrointestinal motility [15–17].

Therefore, the aims of this study were to better describe the distribution and morphology of the EGC in gastric longitudinal muscle myenteric plexus (LMMP) of rats with different periods of diabetic rats by using antibodies against S100B to explore the possible mechanisms of the effects by observing the changes of EGC in the stomach.

## 2. Materials and Methods

### 2.1. Animals

Forty-six adult male Sprague Dawley rats weighing 250–350 g were used in this study. These rats were purchased from the Experimental Animal Center of Tongji Medical College of Huazhong University of Science and Technology (Wuhan, Hubei Province, China). The rats were divided into two large groups randomly including the age-matched control group (CN, *n* = 10) and diabetes group (DM, *n* = 36). The DM group consisted of early diabetes group (EDM, 7–14 days, *n* = 18) and terminal diabetes group (TDM, 56–63 days, *n* = 18); the CN group also contained early control group (ECN, 7–14 days, *n* = 5) and terminal control group (TCN, 56–63 days, *n* = 5). The DM group was then divided into four subgroups (EDM, EDM + SGES, TDM, and TDM + SGES). The times of control groups were the same as diabetes groups. Prior to this study, all protocols were approved by the Institutional Animal Care and Use Committee of the University.

### 2.2. Surgical Procedure

After an overnight fast, all rats were deeply anesthetized with 1% Nembuta (40 mg/kg, ip) and the maintenance anesthetic was given whenever necessary. The surgical procedure was performed through a midline incision with small scissors. Two pairs of temporary cardiac pacing wires (United States Surgical, a division of Tyco Healthcare Group LP) were placed on the serosal surface of the greater curvature of the stomach by unabsorbable sutures. One pair in the middle of the stomach was used for stimulation, with the other in the middle between the proximal pair and the pylorus for recording. The electrodes in each pair were 0.3 cm apart. The insulated connecting wires were subcutaneously brought out to the back of the neck for connection to the recorder or the stimulation equipment. The abdominal wall and skin were then closed in a simple interrupt pattern, and the study was initiated after the rats had fully recovered from the operation, about 10–14 days afterward. This procedure was applied in all groups of rats.

### 2.3. Induction of Diabetes

After a complete recovery from the surgery, diabetes was induced in overnight fasted rats by a one-time intraperitoneal injection of streptozotocin (STZ, 55 mg/kg, Sigma, USA) dissolved in a 1.5 mL citric acid buffer (PH 4.5 Sigma, USA). The control group rats were injected with 1.5 mL citric acid buffer. The blood glucose level was examined one week after STZ injection by cutting off the tip of the tail. Animals exhibiting blood glucose levels more than 16.7 mmol/L were considered diabetic; otherwise they were excluded from our study. The blood glucose was measured again on the day before sacrifice.

### 2.4. Recording of Gastric Intrinsic Gastric Slow Waves

Intrinsic gastric slow waves were recorded from the implanted distal serosal electrodes on the greater curvature using a multichannel recorder (Acknowledge 3.7.1, MP100A-CE, Biopac System, Santa Barbara, CA, USA) in all groups before the injection of STZ or vehicle. Before recording, all rats should be quiet for 30 mins; the signals were displayed on a computer monitor and saved on hard disk. The definition of normal gastric slow wave frequency range was 4–6 cycles/min [17]; it was defined as dysrhythmia if the recording outcome was not in this range. Gastric slow waves were recorded again at 7–14-day diabetes and 56–63-day diabetes before and after stimulation, The age-matched control groups were also recorded again at the same time with diabetic groups.

### 2.5. Synchronized Gastric Electrical Stimulation of Dual Pulses

As is known to all, gastric electrical stimulation (GES) with short pulses improves nausea and vomiting in patients with gastroparesis; however, the effects of short pulses GES on gastric emptying or gastric slow waves were limited [18, 19], whereas GES with long pulses can improve gastric motility and gastrointestinal symptoms of gastroparesis [20]. In this study, we used a novel method, which is called SGES; each stimulus was composed of long pulses (300 ms) followed with five short pulses (width of 0.33 ms, frequency of 100 HZ) and amplitude of 4 mA. Each stimulus was synchronized with the peak of intrinsic gastric slow wave. This new method which has been described earlier is an integrated process with traditional synchronized gastric electrical stimulation, short pulses, and long pulses [21]. An earlier research had demonstrated that SGES can improve vagotomy-induced impairment in gastric accommodation [10]. In our study, SGES was, respectively, performed at early diabetes group (EDM, 7–14 days) and terminal diabetes group (TDM, 567-14–63 days). The SGES contain acute SGES (SGES-60 mins) and chronic SGES (SGES-7 days, 60 mins/day).

### 2.6. Gastric Emptying Tests

After SGES-60 mins or SGES-7 days, gastric emptying was assessed using a validated method as previously described [22]; we modified this method slightly in our study. The rats were fasted for 12 h before the gastric emptying tests. Methylcellulose was dispersed in water at 80°C at a final concentration of 1.5% under continuous stirring. The phenol red (50 mg/mL), used as a nonabsorbable marker, was added. A volume of 2 mL of the phenol red solution was given orally into the stomach through a stainless steel tube. Thirty minutes after stomach perfusion, the animal was euthanized immediately by decapitation. The stomach was removed with the pylorus and the gastroesophageal junction clamped. Then it was cut open and its contents were homogenized in 20 mL distilled water; then 20 mL NaOH (0.5 mol/L) was added and stirred to uniformity. The mixture was kept for 60 mins at room temperature. Afterward, 5 mL of supernatant was added to a test tube with 0.5 mL trichloroacetic acid solution (20% w/v) to precipitate the proteins. After centrifugation (3500 r for 10 mins), the supernatants were analyzed at the end of the study using a spectrophotometer at 560 nM. Meanwhile, a volume of 2 mL of the phenol red solution was added to beaker; subsequently, 18 mL distilled water, 20 mL NaOH (0.5 mol/L), and 4 mL trichloroacetic acid solution (20% w/v) were added successively. The absorbance of the standard sample was measured too. Gastric emptying rate was calculated according to the following equation:

(1) gastric  emptying  rate  (GER)  =1−amount  of  phenol  red  recovered  from  the  stomachaverage  amount  of  phenol  red  recovered  from  the  standard  stomach                                                                                    ×100%.

### 2.7. Electron Microscopy

Antrum of stomach tissue was fixed for 2 h in 2.5% glutaraldehyde (PH: 7.4) and rinsed with 0.1 M phosphate buffer and then fixed with 1% osmic acid. After a series of dehydration, embedding, sectioning, and polymerization, ultrathin sections were cut parallel to either the circular or the longitudinal muscle layers with ultramicrotome (Leica, Ultracut, UCT, Germany) and stained with uranium acetate. These sections were examined with a transmission electron microscope (Tecnai G^2^ 12, FEI Company, Eindhoven, The Netherlands) and photographed for recording.

### 2.8. Immunohistochemistry

This method was used for evaluating the expression of S100B in EGC. After measuring gastric emptying, the rats were sacrificed by cervical dislocation. A median abdominal incision was made to expose the abdominal cavity and the whole stomach was harvested. The specimens of stomach were washed with PBS (PH = 7.4), and they were cut into small segments with a size of approximately 5 mm; then stomach samples were fixed in 4% paraformaldehyde for 4–6 hours at room temperature. Thereafter, tissue blocks were embedded in paraffin; the paraffin-embedded tissue samples were sliced into 5 *μ*m sections with a microtome (Leitz-1512, Germany). Following dewaxing, hydration, and antigen retrieval, sections were then, respectively, treated with primary antibody (rabbit anti-rat S100B antibody, 1 : 250, Abcam, UK) at 4°C overnight and secondary antibody (goat anti-rabbit IgG) at room temperature for 30 mins and stained with haematoxylin and then observed under an optical microscope (Olympus, Tokyo, Japan).

### 2.9. Detection of Rat S100B mRNA by RT-PCR

Real-time polymerase chain reaction (RT-PCR) was used to measure the expression levels of S100B using GAPDH as internal control. Total RNA was isolated using TRIzol reagent (Invitrogen, USA). The obtained RNA samples were reverse transcribed according to instructions. Primer sequences used were S100B forward: 5′-GAGCAGGAAGTGGTGGACAAA-3′, S100B reverse: 5′-CACTCCCCATCCCCATCTT-3′, GAPDH forward: 5′-GTATGACTCTACCCACGGCAAGT-3′, and GAPDH reverse: 5′-TTCCCGTTGATGACCAGCTT-3′. The amplification of S100B and GAPDH fragments was performed using the SYBR Green PCR master mix. A 10 *μ*L PCR reaction volume was containing 5 *μ*L SYBR green reaction mix (Invitrogen, USA), 0.5 *μ*L sense and antisense primers, and 1 *μ*L cDNA. The PCR reaction conditions were 95°C for 10 mins followed by 40 cycles of 95°C for 15 s and 60°C for 1 min. All reactions were performed using the ABI-StepOne Real-Time System (Applied Biosystems, USA). Dissociation curve analysis confirmed the amplification of primer specific products. Relative change in gene expression was determined using the 2^−ΔΔCt^ method [23].

### 2.10. Statistical Analysis

Results are presented as means ± SD. Paired Student's *t*-test was used to compare the blood glucose and weight between the groups before and after injection of STZ. ANOVA was performed to investigate the effect of diabetes on gastric emptying, S100B expression, and the efficacy of SGES. Statistical significance was accepted as *P* < 0.05. All calculations were performed with a commercially available program (SPSS for Windows, version 17.0).

## 3. Results

### 3.1. Development of Diabetes

One week after STZ injection, all rats of diabetes groups presented polydipsia, polyuria, polyphagia. The random blood glucose level of the experimental group always more than 16.7 mmol/L, but the control group was in the normal range. Eight weeks later, the terminal diabetic rats became languid and extreme emaciation. The hair was withered without gloss. Diabetic rats' models were induced successfully in our study (Table 1).

### 3.2. Gastric Slow Waves

**Figure 1:**
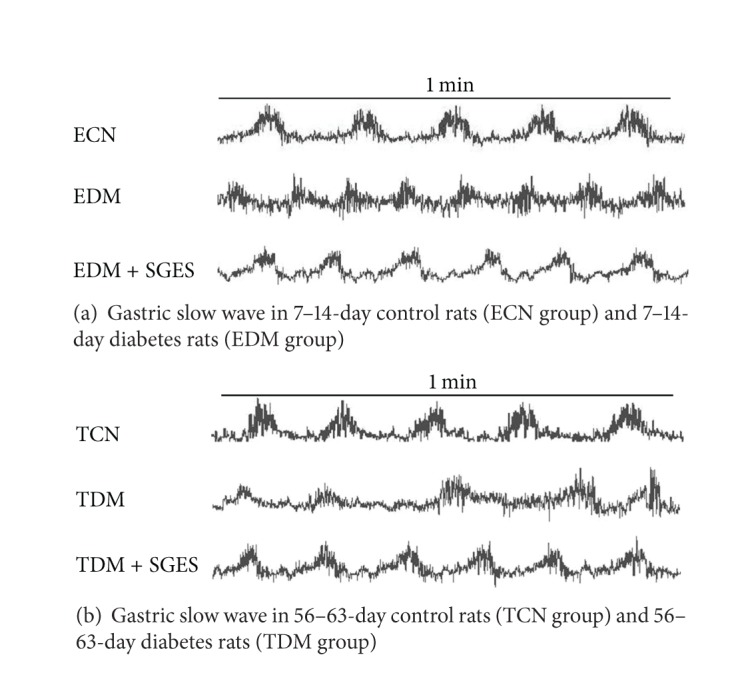
Gastric slow waves in ECN, EDM, TCN, and TDM. The impaired slow waves of TDM can be observed from above recording. While the rhythm in EDM was normal, the frequency of gastric slow waves increased; SGES normalized the gastric slow waves in EDM and TDM. Note: ECN: early control (7–14 days), EDM: early diabetes (7–14 days), TCN: terminal control (56–63 days), and TDM: terminal diabetes (56–63 days). SGES: synchronized gastric electrical stimulation of dual pulses.

The normal slow waves at baseline were shown in Figure 1. One week later after STZ injection, the frequency of slow waves increased at 7–14-day diabetes rats (EDM group). Impairment and rhythm disorder in gastric slow waves were observed in 56–63-day diabetes rats (TDM group).

### 3.3. Gastric Emptying Rates

Gastric emptying rate was significantly delayed in TDM (39.0 ± 7.9% versus 51.8 ± 4.2%, *P* < 0.05). It became faster in EDM (72.0 ± 11.5% versus 58.2 ± 5.4%, *P* < 0.05) than the corresponding control groups. The gastric emptying rate in TCN was a little lower than that in ECN (51.8 ± 4.2% versus 58.2 ± 5.4%, *P* = 0.073). There were statistically significant differences between the control groups and the test groups.

### 3.4. Effect of Synchronized Dual Pulse Gastric Electrical Stimulation on Gastric Emptying

**Figure 2:**
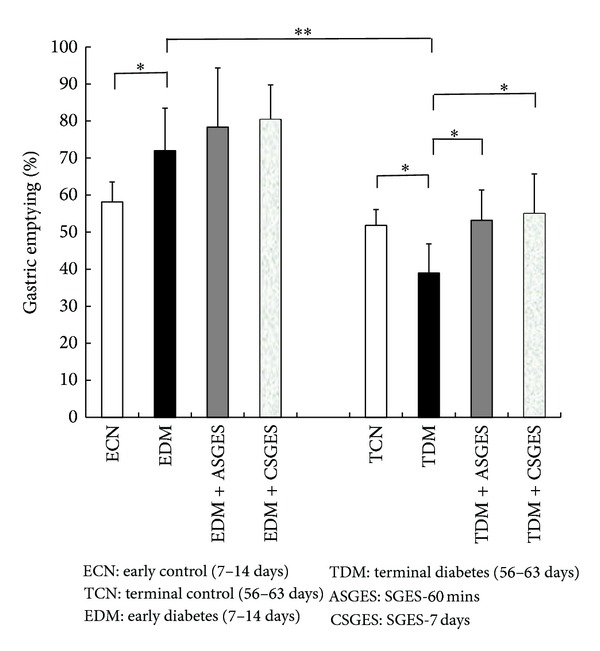
Effect of SGES on gastric emptying. The gastric emptying rates were significantly different between control groups and diabetic groups. Gastric emptying rate was faster in EDM than the age-matched control group but slower in TDM. ASGES and CSGES accelerated gastric emptying in TDM significantly but only had a little effect on EDM. There were no significant differences between EDM and EDM with SGES. Data are presented as means ± SD. *P < 0.05, **P < 0.01.

The acute and chronic synchronized gastric electrical stimulation of dual pulses improved the delay of gastric emptying in TDM group (SGES-60 mins: 39.0 ± 7.9% versus 53.2 ± 8.2%, *P* < 0.05; SGES-7 days: 39.0 ± 7.9% versus 55.03 ± 10.7%, *P* < 0.05), and the gastric emptying of EDM was enhanced a little as shown in Figure 2, but there were no significant differences between EDM and EDM + SGES group (SGES-60 mins: 72.0 ± 11.5% versus 78.3 ± 16.0%, *P* > 0.05; SGES-7 days: 72.0 ± 11.5% versus 81.0 ± 9.4%, *P* > 0.05). The effect of chronic stimulation was superior to acute stimulation. In addition, this new gastric electrical stimulation normalized the gastric slow waves, as shown in Figure 1.

### 3.5. Effect of Synchronized Gastric Electrical Stimulation of Dual Pulses on EGCs

#### 3.5.1. Ultrastructure of EGC

**Figure 3:**
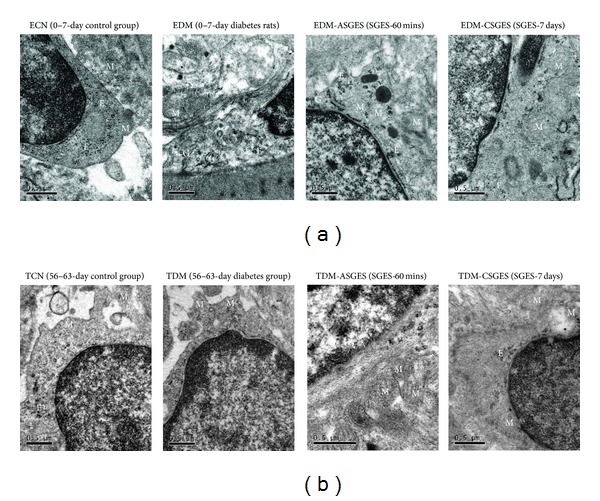
(a) Ultrastructure images of EGC in rat myenteric plexus of ECN, EDM, EDM + ASGES, and EDM+CSGES. Smooth endoplasmic reticulum (letter E), Golgi apparatus (letter G), mitochondria (letter M), and filaments are abundant in cytoplast of ECN and EDM. The number of mitochondria and filaments increased after ASGES and CSGES. And lysosome can be observed in EDM after ASGES. (b) Ultrastructure images of EGC in TCN, TDM, TDM + ASGES, and TDM + CSGES. Mild vacuolization of mitochondria can be observed in cytoplast of TCN. Dilation of endoplasmic reticulum and swelling of mitochondria in cytoplast can be observed in TDM and the filaments decreased seriously. The number of mitochondria and filaments increased after ASGES and CSGES. Note: ASGES: SGES-60 mins; CSGES: SGES-7 days, 60 mins/day.

EGC were mainly located in myenteric plexus of the stomach. In the ECN and EDM, the electron microscope revealed that the smooth endoplasmic reticulum, Golgi apparatus, mitochondria, and filaments are abundant in cytoplast of EGC. While the mild vacuolization of mitochondria can be observed in cytoplast in TCN, the number of EGC significantly reduced in TDM. Dilation of endoplasmic reticulum and swelling of mitochondria in cytoplast can be observed in TDM and the filaments decreased seriously. Comparing with the age-matched diabetic groups, the number of mitochondria and filaments increased after SGES-60 mins and SGES-7 days both in EDM and TDM groups (Figure 3).

#### 3.5.2. Immunohistochemistry of S100B

**Figure 4:**
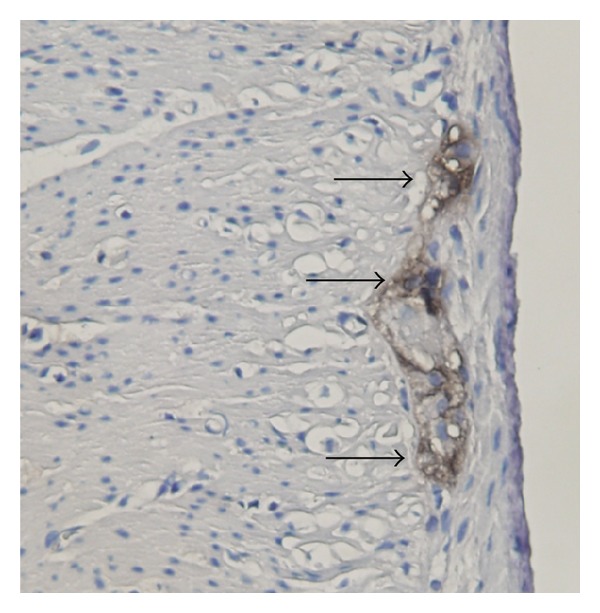
EGC in the rat myenteric plexus of the stomach (protein gene product S100B immunohistochemistry, ×40); dark brown region (black arrows) is EGC of myenteric nerve plexus. EGC are gathered together and form a cell network. Enteric neurons are packed around tightly by EGC.

**Figure 5:**
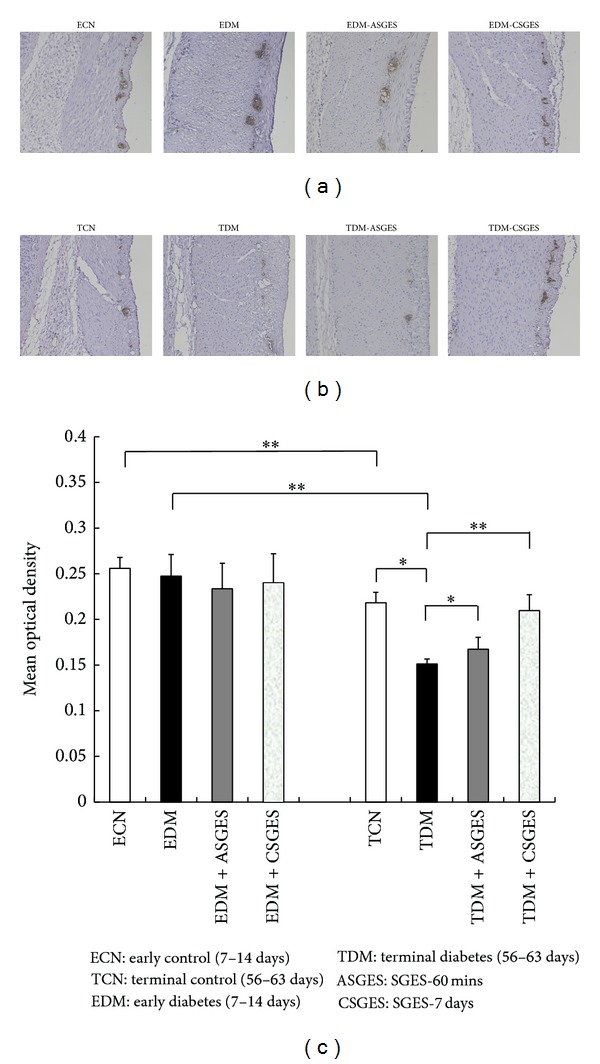
Immunohistochemical staining of S100B in the enteric plexus of stomach. Tissues with brown deposits were positive reaction. (a) S100B expression in ECN, EDM, EDM + ASGES, and EDM + CSGES. (b) S100B expression in TCN, TDM, TDM + ASGES, and TDM + CSGES. (c) Semiquantification of S100B-positive cells in each group. There were no significant differences between ECN and EDM. The expression of S100B in TDM and TCN groups was lower than in EDM and ECN groups, respectively. ASGES and CSGES had little effect on the expression of S100B in EDM. Comparing with TDM group, the immunopositive stain was decreased significantly in TDM group. But the immunopositive stain significantly increased after ASGES and CSGES. Data are presented as means ± SD. *P < 0.05, **P < 0.01.

Figures 4 and 5 showed the immunohistochemical staining of S100B in gastric myenteric plexus. Statistical analysis demonstrated that S100B expression was significantly decreased in TDM compared with the corresponding control group (0.15 ± 0.01 versus 0.22 ± 0.01, *P* < 0.01). There were no significant differences of S100B immunostaining observed between EDM and the corresponding control group (0.26 ± 0.01 versus 0.25 ± 0.02, *P* > 0.05). Besides, we found that the expression of S100B was also decreased in TCN compared with ECN due to the growth of age (0.22 ± 0.01 versus 0.26 ± 0.01, *P* < 0.01). The immunopositive stain in TDM significantly increased after SGES-60 mins and SGES-7 days (SGES-60 mins: 0.15 ± 0.01 versus 0.17 ± 0.01, *P* < 0.05; SGES-7 days: 0.15 ± 0.01 versus 0.21 ± 0.02, *P* < 0.05); but SGES-60 mins and SGES-7 days had little effect on the expression of S100B in EDM (SGES-60 mins: 0.25 ± 0.02 versus 0.23 ± 0.03, *P* > 0.05; SGES-7 days: 0.25 ± 0.02 versus 0.24 ± 0.03, *P* > 0.05).

#### 3.5.3. S100B mRNA Expression Analysis

**Figure 6:**
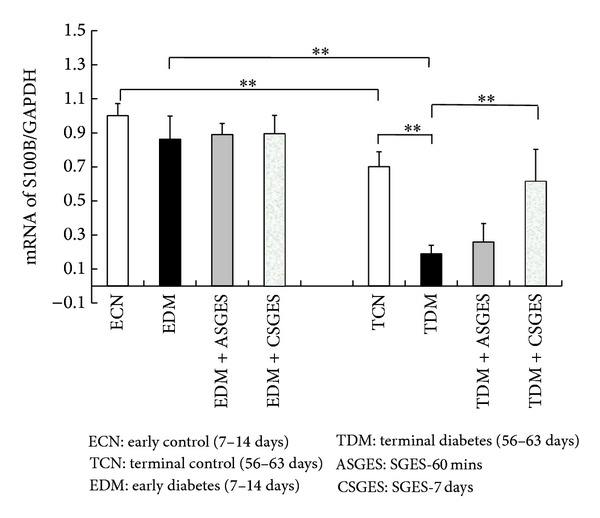
mRNA values of S100B in the enteric plexus of stomach. The mRNA expression of S100B was decreased with the course of diabetes, especially in TDM. But there were no differences between ECN and EDM. Furthermore, both mRNA expressions of S100B in TDM and TCN were significantly decreased compared with the age-matched early group. The expression of S100B mRNA in TDM increased both after ASGES and CSGES compared with TDM; statistical analysis showed significant differences between CSGES group and TDM group, but no statistical differences were observed between ASGES and TDM. Data are presented as means ± SD. *P < 0.05, **P < 0.01.

The mRNA expression of S100B was shown in Figure 6. RT-PCR studies showed that the change trends of S100B were decreased with the course of diabetes, especially in the TDM (0.19 ± 0.05 versus 0.70 ± 0.09, *P* < 0.01). But there were no differences between EDM and ECN (0.86 ± 0.14 versus 1.00 ± 0.07, *P* > 0.05). The S100B mRNA expression of TDM and TCN was significantly decreased compared to EDM (0.19 ± 0.05 versus 0.86 ± 0.14, *P* < 0.01) and ECN (0.70 ± 0.09 versus 1.00 ± 0.07, *P* < 0.01), respectively. Compared with age-matched control group, the expression of S100B mRNA increased both in SGES-60 mins and SGES-7-day group, and statistical analysis showed significant differences between SGES-7 days and TDM, but no differences between SGES-60 mins and TDM.

## 4. Discussion

In this current study, the model of diabetes mellitus in rats was induced successfully by injection of streptozotocin (STZ). We found that gastric emptying rate was delayed in 56–63-day diabetes rats, but that in 7–14-day diabetes rats increased. The number of EGC in 56–63-day diabetes rats decreased and the ultrastructure changed. Additionally, we demonstrated the age-associated loss of EGC between 7–14-day control rats and 56–63-day control rats. SGES was able to not only accelerate gastric emptying in 56–63-day diabetes rats and 7–14-day diabetes rats but also normalize gastric slow waves. We also demonstrated that the expression of S100B protein which was used frequently as EGC marker decreased in 56–63-day diabetes rats. However, this marker expression increased after SGES and the ultrastructure of EGC was partially restored whether acute stimulation or chronic stimulation. The effect of SGES on gastric emptying may be associated with EGC activation.

Gastroparesis is a disorder in which the gastric emptying is delayed without mechanical obstruction [1]. The etiology of diabetes gastroparesis is diverse and the pathogenesis is not well illustrated. However, in many cases, the condition results from damage to the vagus nerve that controls gastric muscles due to long-term and high level of blood glucose level in blood [24]. Gastric emptying is a coordinated function under the domination of ENS by intense peristaltic contractions in the antrum. If the vagus nerve is damaged or severed, muscles of the stomach and intestine do not work normally or do not work at all. Food then moves slowly or stops moving through the digestive tract, thus leading to delayed gastric emptying. But previous researches have showed that delayed gastric emptying returned to normal one month later after truncal vagotomy; this finding implied that neural pathways other than the truncal vagus compensated impaired motility functions [11].

As is known to all, the damage of ENS always occurred both in diabetic patients and animal models [25, 26]. The ENS is now recognized as a very complex and huge structure; it exerts a profound influence on all digestive processes, namely, immune response, detecting nutrients, motility, microvascular circulation, intestinal barrier function, and epithelial secretion of fluids, ions, and bioactive peptides [27]. The ENS referred to as the “second brain” derives from the neural crest and consists mainly of EGC, ICC, and neurons, of which the EGC are main components [28]. During the past years, a growing number of evidences had indicated that gastrointestinal tract motility disorders, such as gastroparesis and constipation, often accompany diabetes. In some patients with severely slow-transit constipation, the loss of EGC but not of ICC and enteric neurons was documented in the terminal ileum [8, 29]; in addition, EGC abnormalities are found in the majority of patients with gastroparesis [30]. All these studies suggest that the reduction of EGC might be involved in mechanism of diabetic delayed gastric emptying.

In gastrointestinal tract, the EGC mainly distributed in submucosal and myenteric plexuses and surrounded enteric neurons (Figure 4). The morphology of mature EGC is similar to astrocyte in the central nervous system (CNS). Traditional view suggested that EGC have only mechanical support for surrounding neurons; as gradual progress of the research in this field, the EGC have more articulate and complex nature involved in regulation of gastrointestinal tract motility and maintenance of intestinal homeostasis [31]. The S100B protein is thought to be localized in these cells exclusively. This kind of protein is the marker in identifying EGC [32]. Our observation showed that the expression quantity of S100B in diabetic rats induced by STZ was associated with diabetes duration. The EGC in the myenteric plexus of stomach were decreased in 56–63-day diabetes rats but not in 7–14-day diabetes rats, and, compared with that in 7–14-day control rats, the expression of S100B in 56–63-day control rats also decreased. This reduction might be related to a reduction in the expression of neurotrophins responsible for promoting the survival and maintenance of neurons [33]. The changes in the EGC are likely to play an important role in alteration in motility function of the gastrointestinal tract. In addition, animal experiments have demonstrated that the EGC are related to gastrointestinal tract motility [34]. While the structure and function of EGC changed, gastrointestinal motility and gastric emptying would be delayed [35, 36].

S100B belongs to the S100 protein family; it is a kind of Ca^2+^ binding protein. S100B protein produced the best results in identifying EGC. This protein regulates cytoskeletal structure and function and calcium homeostasis in EGC. It is thought to be exclusively localized in these cells. The S100B in brain can promote neuronal survival, increase free Ca^2+^ levels in vitro, and participate in Ca^2+^ waves' start and propagation, but the date on EGC is limited [37, 38]. Nevertheless, several researches have demonstrated the intercellular Ca^2+^ waves in cultured EGC and revealed the intercellular signaling mechanisms [39, 40]. These Ca^2+^ waves can be initiated by stimulation of various neurotransmitter receptors in the plasma membrane. Furthermore, these studies found the phospholipase C, inositol trisphosphate, and ATP playing an important role in propagating Ca^2+^ waves. Our finding showed that the S100B expression significantly increased after SGES-60 mins and SGES-7 days in 56–63-day diabetes rats but not in 7–14-day diabetes; the effect of SGES on gastric emptying may be related to active EGC. A recent study in human EGC confirms that S100B is able to integrate toll-like receptor signaling, which induces the activation of EGC [41]. The active EGC can release ATP which participate and mediate the Ca^2+^ waves propagating in EGE-to-EGC and EGC-to-neighbouring cells via gap junctions throughout the EGC networks. In addition, a previous study had proved that the ATP released from EGC may provide a feedback mechanism for pacemaker activity of ICC in the intestine [42].

However, gastric emptying in 7–14-day diabetes rats increased; the number of EGC has no obvious change in our study. The pathogenesis of accelerated gastric emptying in rats with early diabetes is heterogeneous and the mechanism remains poorly understood. But it has been reported that fasting ghrelin plasma concentrations were elevated in the early stage of diabetes compared with those rats without diabetes [43]. The elevated ghrelin enhanced the incidence of postprandial antropyloric coordination. In addition, there was a study indicating that the accelerated gastric emptying was related to human islet amyloid polypeptide (hIAPP). hIAPP is a hormone synthesized and secreted by the pancreatic *β*-cells; monomeric hIAPP was suggested to have potent inhibitory effects on gastric emptying [44]. Even if there is no enteric neuropathy in the early diabetes, the hIAPP was deficient due to the impaired pancreatic *β*-cell. Some studies showed that the decreased serum leptin may also participate in the accelerated gastric emptying of early diabetes. Meanwhile, Itoh et al. indicated that unmyelinated fibers size of the vagus in nerves decreased significantly in the spontaneously diabetic Chinese hamster, resulting in accelerated gastric emptying by diminishing vagovagal inhibitory reflex pathway [45]. Thus we speculated that the gastric emptying had no significant relevance with EGC in 7–14-day diabetes rats and the accelerated gastric emptying rate may be explained by an adaptation to increased glucose absorption through the gastrointestinal tract and the acute changes in the blood glucose concentration.

To our surprise, both SGES-60 mins and SGES-7 days have no obvious effect on the expression of S100B in 7–14-day diabetes rats. The ghrelin plasma concentrations enhanced at 7–14-day diabetes rats as we mentioned above. And Lopez et al. have reported that ghrelin is capable of decreasing serum concentration of the astrocytic protein S100B in brain [46]. When SGES was taken, the increased S100B may be offset by the increased ghrelin concentrations at early stage of diabetes rats. These findings gave rise to the hypothesis that this phenomenon may be related to enhanced ghrelin, although the date in gastrointestinal tract is limited. Another important finding of our study was the age-associated loss of EGC. We noted that the expression of S100B decreased in 56–63-day control rats compared with 7–14-day control rats. And the gastric emptying of 56–63-day control rats was also reduced a little. This finding has been reported several years ago and it demonstrated that the loss of EGC was proportional to neuronal death [47]. The loss of EGC was likely related to neurodegeneration and might be expected to increase the susceptibility of delayed gastric emptying in 56–63-day control rats. In addition, the vagal and sympathetic extrinsic nerves dystrophia, the loss of ICC, and the increased visceral sensitivity may be major factors. Further research on EGC would be helpful to understand the gastrointestinal motility disorders with aging.

In summary, SGES not only normalizes gastric slow waves in 7–14-day diabetes rats and 56–63-day diabetes rats but also accelerates gastric emptying in 56–63-day diabetes rats. The effects of SGES on S100B expression in 7–14-day diabetes rats are little which indicates the other factors such as the fact that ghrelin is involved in S100B. Meanwhile, the impaired EGC can be partly restored which makes us have a good understanding of the plasticity of ENS. This study has highlighted a potential role of EGC in the pathophysiology of diabetic gastroparesis. Further studies such as patch clamp technique and calcium imaging experiment in EGC are needed to gain a better understanding of the signaling mechanism of EGC activation in the action of SGES.

## Figures and Tables

**Table 1:** Blood glucose and body weight in controls and diabetes mellitus rats.

Group	*n*	Body weight, g		Blood glucose, mmol/L	
		Before injection	After injection	Before injection	After injection
ECN	5	276.5 ± 25.16	312.4 ± 32.35	6.92 ± 0.24	9.92 ± 2.11
EDM	6	243.0 ± 39.79	222.7 ± 22.11^b^	8.15 ± 2.63	26.00 ± 4.46^a,b^
EDM + SGES	12	269.2 ± 26.66	228.5 ± 25.48^a,b^	10.14 ± 2.59	26.65 ± 4.51^a,b^
TCN	5	254.4 ± 31.60	437.0 ± 35.09	7.82 ± 1.47	7.68 ± 1.59
TDM	6	256.8 ± 20.88	197.0 ± 20.06^a,b^	8.78 ± 1.51	26.87 ± 3.72^a,b^
TDM + SGES	12	280.6 ± 25.17	208.6 ± 16.61^a,b^	9.07 ± 3.15	26.57 ± 4.22^a,b^

Data are presented as means ± SD. ECN: early control (7–14 days), EDM: early diabetes (7–14 days), TCN: terminal control (56–63 days), and TDM: terminal diabetes (56–63 days). SGES: synchronized gastric electrical stimulation of dual pulses. ^a^ *P* < 0.001 versus baseline, ^b^ *P* < 0.001 versus control.

## References

[1] Parkman HP, Hasler WL, Fisher RS (2004). American Gastroenterological Association technical review on the diagnosis and treatment of gastroparesis. *Gastroenterology*.

[2] Enck P, Rathmann W, Spiekermann M (1994). Prevalence of gastrointestinal symptoms in diabetic patients and non-diabetic subjects. *Zeitschrift fur Gastroenterologie*.

[3] Soykan I, Sivri B, Sarosiek I, Kiernan B, Mccallum RW (1998). Demography, clinical characteristics, psychological and abuse profiles, treatment, and long-term follow-up of patients with gastroparesis. *Digestive Diseases and Sciences*.

[4] Revicki DA, Camilleri M, Kuo B (2009). Development and content validity of a gastroparesis cardinal symptom index daily diary. *Alimentary Pharmacology and Therapeutics*.

[5] Rodrigues MLC, Motta MEFA (2012). Mechanisms and factors associated with gastrointestinal symptoms in patients with diabetes mellitus. *Jornal de Pediatria*.

[6] Soffer EE (2012). Gastric electrical stimulation for gastroparesis. *Journal of Neurogastroenterology and Motility*.

[7] Gallas S, Fetissov SO (2011). Ghrelin, appetite and gastric electrical stimulation. *Peptides*.

[8] Bortolotti M (2011). Gastric electrical stimulation for gastroparesis: a goal greatly pursued, but not yet attained. *World Journal of Gastroenterology*.

[9] Song G, Chen JDZ (2007). Synchronized gastric electrical stimulation improves delayed gastric emptying in nonobese mice with diabetic gastroparesis. *Journal of Applied Physiology*.

[10] Chen J, Koothan T, Chen JDZ (2009). Synchronized gastric electrical stimulation improves vagotomy-induced impairment in gastric accommodation via the nitrergic pathway in dogs. *American Journal of Physiology—Gastrointestinal and Liver Physiology*.

[11] Howlett PJ, Ward AS, Duthie HL (1974). Gastric emptying after vagotomy. *Proceedings of the Royal Society of Medicine*.

[12] Du F, Wang L, Qian W, Liu S (2009). Loss of enteric neurons accompanied by decreased expression of GDNF and PI3K/Akt pathway in diabetic rats. *Neurogastroenterology and Motility*.

[13] Bassotti G, Villanacci V, Cathomas G (2006). Enteric neuropathology of the terminal ileum in patients with intractable slow-transit constipation. *Human Pathology*.

[14] Gomes P, Chevalier J, Boesmans W (2009). ATP-dependent paracrine communication between enteric neurons and glia in a primary cell culture derived from embryonic mice. *Neurogastroenterology and Motility*.

[15] Chalazonitis A, D’Autréaux F, Pham TD, Kessler JA, Gershon MD (2011). Bone morphogenetic proteins regulate enteric gliogenesis by modulating ErbB3 signaling. *Developmental Biology*.

[16] Bassotti G, Villanacci V, Fisogni S (2007). Enteric glial cells and their role in gastrointestinal motor abnormalities: introducing the neuro-gliopathies. *World Journal of Gastroenterology*.

[17] Bassotti G, Villanacci V, Antonelli E, Morelli A, Salerni B (2007). Enteric glial cells: new players in gastrointestinal motility?. *Laboratory Investigation*.

[18] Liu J, Qiao X, Micci M, Pasricha PJ, Chen JDZ (2004). Improvement of gastric motility with gastric electrical stimulation in STZ-induced diabetic rats. *Digestion*.

[19] Chen JDZ, Qian L, Ouyang H, Yin J (2003). Gastric electrical stimulation with short pulses reduces vomiting but not dysrhythmias in dogs. *Gastroenterology*.

[20] Lin Z, Forster J, Sarosiek I, McCallum RW (2004). Treatment of diabetic gastroparesis by high-frequency gastric electrical stimulation. *Diabetes Care*.

[21] McCallum RW, Chen JDZ, Lin Z (1998). Gastric pacing improves emptying and symptoms in patients with gastroparesis. *Gastroenterology*.

[22] Scarpignato S, Capovilla T, Bertaccini G (1980). Action of caerulein on gastric emptying of the conscious rat. *Archives Internationales de Pharmacodynamie et de Therapie*.

[23] Livak KJ, Schmittgen TD (2001). Analysis of relative gene expression data using real-time quantitative PCR and the 2^−ΔΔCt^ method. *Methods*.

[24] Yin J, Chen J, Chen JDZ (2010). Ameliorating effects and mechanisms of electroacupuncture on gastric dysrhythmia, delayed emptying, and impaired accommodation in diabetic rats. *American Journal of Physiology—Gastrointestinal and Liver Physiology*.

[25] Brock C, Graversen C, Frøkjaer JB (2012). Peripheral and central nervous contribution togastrointestinal symptoms in diabetic patients with autonomic neuropathy. *European Journal of Pain*.

[26] Bagyánszki M, Bódi N (2012). Diabetes-related alterations in the enteric nervous system and its microenvironment. *World Journal of Diabetes*.

[27] Nezami BG, Srinivasan S (2010). Enteric nervous system in the small intestine: pathophysiology and clinical implications. *Current Gastroenterology Reports*.

[28] Wang X, Chan AKK, Sham MH, Burns AJ, Chan WY (2011). Analysis of the sacral neural crest cell contribution to the hindgut enteric nervous system in the mouse embryo. *Gastroenterology*.

[29] Bassotti G, Villanacci V, Maurer CA (2006). The role of glial cells and apoptosis of enteric neurones in the neuropathology of intractable slow transit constipation. *Gut*.

[30] Grover M, Farrugia G, Lurken MS (2011). Cellular changes in diabetic and idiopathic gastroparesis. *Gastroenterology*.

[31] Rühl A (2005). Glial cells in the gut. *Neurogastroenterology and Motility*.

[32] Ferri GL, Probert L, Cocchia D (1982). Evidence for the presence of S-100 protein in the glial component of the human enteric nervous system. *Nature*.

[33] Pereira RVF, Tronchini EA, Tashima CM, Alves EPB, Lima MM, Zanoni JN (2011). L-glutamine supplementation prevents myenteric neuron loss and has gliatrophic effects in the ileum of diabetic rats. *Digestive Diseases and Sciences*.

[34] Qi R, Yang W, Chen J (2013). Role of enteric glial cells in gastric motility in diabetic rats at different stages. *Journal of Huazhong University of Science and Technology. Medical Sciences*.

[35] Nasser Y, Fernandez E, Keenan CM (2006). Role of enteric glia in intestinal physiology: effects of the gliotoxin fluorocitrate on motor and secretory function. *American Journal of Physiology—Gastrointestinal and Liver Physiology*.

[36] Aubé A-C, Cabarrocas J, Bauer J (2006). Changes in enteric neurone phenotype and intestinal functions in a transgenic mouse model of enteric glia disruption. *Gut*.

[37] Chow S, Yu D, Macdonald CL, Buibas M, Silva GA (2010). Amyloid *β*-peptide directly induces spontaneous calcium transients, delayed intercellular calcium waves and gliosis in rat cortical astrocytes. *ASN Neuro*.

[38] Verkhratsky A, Butt A (2007). *Glial Neurobiology*.

[39] Zhang W, Segura BJ, Lin TR, Hu Y, Mulholland MW (2003). Intercellular calcium waves in cultured enteric glia from neonatal guinea pig. *Glia*.

[40] Broadhead MJ, Bayguinov PO, Okamoto T, Heredia DJ, Smith TK (2012). Ca^2+^ transients in myenteric glial cells during the colonic migrating motor complex in the isolated murine large intestine. *The Journal of Physiology*.

[41] Turco F, Sarnelli G, Cirillo C (2014). Enteroglial-derived S100B protein integrates bacteria-induced Toll-like receptor signalling in human enteric glial cells. *Gut*.

[42] Burnstock G, Lavin S (2002). Interstitial cells of Cajal and purinergic signalling. *Autonomic Neuroscience: Basic and Clinical*.

[43] Ariga H, Imai K, Chen C, Mantyh C, Pappas TN, Takahashi T (2008). Does ghrelin explain accelerated gastric emptying in the early stages of diabetes mellitus?. *American Journal of Physiology—Regulatory Integrative and Comparative Physiology*.

[44] Young A (2005). Inhibition of gastric emptying. *Advances in Pharmacology*.

[45] Itoh H, Yoneda M, Tamori K (1995). Rapid gastric emptying and pathological changes of vagus nerve in the spontaneously diabetic Chinese hamster. *Diabetes Research and Clinical Practice*.

[46] Lopez NE, Krzyzaniak MJ, Blow C (2012). Ghrelin prevents disruption of the blood-brain barrier after traumatic brain injury. *Journal of Neurotrauma*.

[47] Phillips RJ, Kieffer EJ, Powley TL (2004). Loss of glia and neurons in the myenteric plexus of the aged Fischer 344 rat. *Anatomy and Embryology*.

